# Measuring sedentary behavior using waist- and thigh-worn accelerometers and inclinometers – are the results comparable?

**DOI:** 10.1177/1759720X221079256

**Published:** 2022-03-15

**Authors:** Tobias Kalisch, Christoph Theil, Georg Gosheger, Thomas Ackmann, Isabell Schoenhals, Burkhard Moellenbeck

**Affiliations:** Department of Orthopedics and Tumor Orthopedics, Muenster University Hospital, Albert-Schweitzer-Campus 1, 48149 Muenster, Germany; Department of Orthopedics and Tumor Orthopedics, Muenster University Hospital, Muenster, Germany; Department of Orthopedics and Tumor Orthopedics, Muenster University Hospital, Muenster, Germany; Department of Orthopedics and Tumor Orthopedics, Muenster University Hospital, Muenster, Germany; Department of Orthopedics and Tumor Orthopedics, Muenster University Hospital, Muenster, Germany; Department of Orthopedics and Tumor Orthopedics, Muenster University Hospital, Muenster, Germany

**Keywords:** accelerometer, adult, inclinometer, physical activity, sedentary behavior

## Abstract

**Background:**

Objective sensor-based quantification of sedentary behavior is an important tool for planning and evaluating interventions for excessive sedentary behavior in patients with musculoskeletal diseases. Although waist-worn accelerometers are the standard for physical activity (PA) assessment, only thigh-worn inclinometers can clearly distinguish sedentary behavior from any light PA or standing activity.

**Methods:**

In this study, 53 adults (ages 20–85 years) wore two ActiGraph wGT3X-BT monitors, each containing an inclinometer and accelerometer (set for acquisition of slow movements in all three planes), attached to the right waist and thigh for a period of about 4 days. Both monitors recorded total sedentary time and continuous sedentary 10-min bouts by synchronous accelerometry and inclinometry. Differences and correlations between methods and wearing positions were evaluated against participant age, body mass index (BMI), and number of steps taken. Thigh-worn inclinometry was used as reference.

**Results:**

Data from thigh-worn inclinometry and waist-worn accelerometry were highly correlated for total sedentary time [rho = 0.888; intraclass correlation coefficient (ICC) = 0.937] and time in sedentary bouts (rho = 0.818; ICC = 0.848). Nevertheless, accelerometry at the waist underestimated sedentary time by ≈17% (*p* < 0.001) and time in sedentary bouts by ≈54% (*p* < 0.001). A satisfactory concordance thus could be demonstrated only for total sedentary time, based on the Bland–Altmann method (≈96% of data within the limits of agreement). The differences between waist-worn accelerometry and thigh-worn inclinometry did not correlate with age but did correlate with BMI and PA for both sedentary behavior parameters (*r* ⩾ 0.240, *p* ⩽ 0.043).

**Conclusion:**

A waist-worn accelerometer can be used to determine total sedentary time under free-living conditions with sufficient accuracy if the correct settings are chosen. Further investigations are necessary to investigate why short sedentary bouts cannot be reliably assessed.

**Trial registration:**

DRKS00024060 (German Clinical Trials Register)

## Background

Sedentary behavior (SB) is defined as any waking activity performed in a sitting, reclining, or lying posture that does not substantially increase energy expenditure.^1^ Strong evidence shows the negative health consequences of excessive SB with regard to obesity, cardiovascular disease, diabetes, cancer, and overall mortality.^2^ Findings indicate a dose–response relationship between time spent sitting and chronic disease, particularly in older adults, which represent the most sedentary population.^3^ Additional physical activity (PA) does not seem to prevent the negative effects of SB.^4,5^ Consequently, even younger people who spend long periods of time sitting because of their job might not be able to rely on leisure-time exercise to fully compensate for the negative effects of excessive SB. Further research is needed to gain more detailed insights into the relationship between SB and PA, and valid measures of SB are needed to assess its health effects and evaluate interventions designed to reduce this negative health behavior.^6^

Most knowledge about SB is derived from studies relying on self-report, although questionnaires have poor validity because respondents usually underestimate their sedentary time.^7^ Objective methods such as accelerometry (an indirect sensor-based method that deduces motion from measured accelerations), which infer SB through a lack of body movement, tend to overestimate SB compared with questionnaires. The reason is that these devices cannot clearly distinguish sitting from standing or incorporate everyday activities with limited ambulatory movement, such as cooking, cashiering, or working at a hotel reception desk.^8^ Accordingly, a review of studies investigating SB in older adults found large discrepancies between their average daily sedentary times measured with accelerometers (9.4 h/day) and self-reports (5.3 h/day).^9^ Accelerometers have become the standard instrument in determining SB in both laboratory^10^ and free-living conditions,^11^ but are not problem free. Because of the large number of different monitors and mounting and positioning options, and different modes of data processing and evaluation, recommendations are lacking to guide their optimal use in SB research.^12^ In addition, multiple combinations of settings and often-missing information about those settings make it difficult to compare results across studies.

Most of the common accelerometer settings are primarily designed to capture motion (i.e. PA) and not SB. For example, older accelerometers recorded motion along only one body axis (the vertical or Y axis). These algorithms remain in wide use although newer monitors can capture motion in all three planes (X, Y, and Z axes) and process them as vector magnitude (VM, the square root of the sum of the squared accelerations in all three planes, used as a composite measure of acceleration). Unfortunately, little is known about the appropriate thresholds between SB and non-SB using the VM.^10,13^ In addition, to capture SB, filter settings optimized to quantify slow movements should be selected to better differentiate between sitting and light PA. However, because such filters also affect the overall recording of PA, they often are not used because the results would be non-comparable with previous PA studies.^14^

To circumvent technical problems in the acquisition of SB and to obtain more accurate results, inclinometers have been incorporated into some accelerometers. Inclinometers measure the orientation angle of an object with respect to the force of gravity. This measurement is achieved by separate sensors that monitor the effect of gravity on a tiny mass suspended in an elastic support structure. This function can be used to determine the alignment of a specific body part (torso, thigh) in space and guide conclusions about the posture of the entire body, allowing for direct detection of current posture and transitions between postures.^14^ Inclinometry could be of great value for SB research because even light activities, such as standing still (which accelerometers could incorrectly classify as SB), seem to have positive effects on some markers of health,^15^ although these findings are controversial.^16^ Many studies have used accelerometry to indirectly determine SB, but the number of studies using inclinometry is limited.

Among the most used monitors for determining PA as well as SB is the GT3X accelerometer (ActiGraph LLC, Pensacola, FL, USA), which also includes an inclinometer. Other models of this monitor (such as the wGT3X-BT used in the present study) use the same sensor technology to determine PA and SB but have been enhanced with wireless technology for data transmission. Growing evidence indicates that the ActiGraph proprietary inclinometer algorithm is highly accurate in identifying posture when the monitor is attached to the thigh. The reliability of SB detection was reported to range from 86% to 100%, in agreement with direct observation.^17–20^ However, for monitors worn on the waist, previous studies reported a rather poor reliability in detecting SB,^10,17,19,21^ calling its usefulness into question.

To date, only limited data exist on the differences in the detection of SB using waist- and thigh-worn accelerometers and inclinometers under free-living conditions. Thus, the primary purpose of this descriptive, cross-sectional study was to investigate SB detection by means of simultaneously worn wGT3X-BT monitors in adults. The focus was on a direct comparison between the waist-worn accelerometer and the thigh-worn inclinometer. The secondary purpose was to identify possible demographic parameters that could affect the extent of conformity between the methods.

## Methods

### Recruitment and eligibility criteria

A convenience sample of 53 adults volunteered to participate in the study. Participants were recruited *via* word of mouth and e-mail at the City of Remscheid and the surrounding area of North-Rhine Westphalia, Germany. Inclusion criteria were as follows: age ⩾ 18 years to ⩽ 80 years, German language proficiency, and no mobility issues (lower limb amputations, prostheses, walking aids, etc.). Participants who reported any physical or psychological problem limiting their otherwise normal daily routines with respect to SB and PA were excluded from participation.

Ethical approval was obtained by the local ethics committee (Ethik Kommission der Ärztekammer Westfalen-Lippe und der Westfälischen Wilhelms-Universität, ref. no 2021-026-f-S). Since this was not a pharmacological study, the manuscript was written following the STARD protocol (‘Standards for Reporting Diagnostic accuracy studies’) as closely as possible.^22^ Furthermore, the study was conducted according to the principles of the declaration of Helsinki, and all participants provided written informed consent before participation.

### Study design

The investigation was designed as a cross-sectional study. Interested participants were checked for eligibility and provided with the relevant documents. After receipt of written consent to participate in the study and the return of the documents, each participant personally received a set of accelerometers and instructions for their use. Supplementary written instructions also were handed out.

### Objective SB and PA measures

The ActiGraph wGT3X-BT (ActiGraph LLC, Pensacola, FL, USA) is a small (45 × 33 × 15 mm), lightweight (19 g) triaxial accelerometer that can be attached to various body locations including the waist, wrist, ankle, and thigh. Using a proprietary algorithm (firmware v1.9.2), the waist-worn monitor can detect posture (lying, sitting, standing) and non-wear. When the monitor is attached to the thigh, the lying and sitting categories are grouped together. The wGT3X-BT monitors were initialized according to the manufacturer’s specifications to record at a frequency of 100 Hz, and the low-frequency extension (LFE) filter was selected. Participants wore two monitors simultaneously: one on an elastic belt around the right waist (at the intersection of a vertical line between the armpit and the knee) and one on an elastic belt at the midline on the anterior aspect of the right thigh. Each pair of monitors was synchronized in time. Participants were instructed to always wear both monitors and to take them off only for water-based activities and sleeping. Moreover, study eligibility required that participants agree to wear the monitors for a period of at least two consecutive days.

ActiGraph data were downloaded using the manufacturer’s software, ActiLife v6.13.4, and converted into 10-s epoch comma-separated values (CSV) files. Further evaluation was performed using a custom-built MS Excel template. The following waist monitor data were evaluated in 10-s epochs: VM (cpm), steps (#), non-wear (s), sitting (s), and lying (s). Similarly, VM (cpm), steps (#), and sitting/lying (s) were collected from the thigh monitor. Data were analyzed only when the waist-worn inclinometer detected an entire epoch as standing, sitting, or lying down. If the monitor was not worn (‘inclineoff’, i.e. the *z*-axis offset angle θz < 22 degrees and accelerometer activity below six counts per second^23^), the epoch was discarded. To enable methodological comparison, valid wear times were additionally calculated by applying the Choi *et al.*^24^ algorithm (minimum length 90 min, small window length 30 min, spike tolerance 2 min) to the VM data from the waist- and thigh-worn accelerometers.

For each participant, total sedentary time of both monitors was calculated based on accelerometer data (VM ⩽ 150 cpm^25^) and inclinometer data (sitting times and lying times summed) from both monitors. In addition, continuous sedentary times in 10-min intervals (bouts) were calculated from data of both monitors, with an allowed ‘drop time’ of 2 min, in which the VM was allowed to be above the threshold without the bout being discarded. These sedentary 10-min bouts are the counterpart of continuous times in increased PA (i.e. bouts of moderate-to-vigorous PA), which have been repeatedly shown to have beneficial effects on health.^26^

The first and last 30 s of each recording were excluded from the analyses to protect against potential imperfect time synchronization and transition between activities. In addition, basic demographic data including age, sex, height, weight, and body mass index (BMI) were recorded.

### Statistical analysis

Descriptive analyses were performed on the demographic variables and on average wear time of the sample. The epoch-based accelerometer and inclinometer data were outlier-corrected (boxplot-method), tested for normal distribution (Shapiro–Wilk test), and presented as means (M) and standard deviation (SDs) [or 95% confidence intervals (CIs)] or as medians (Mdns) and 25% to 75% interquartile ranges (IQRs). Subsequent analyses were performed in a two-step process comprising (a) a comparison of data and (b) an investigation of the association of data following the approach of Mandigout *et al.*,^27^ as well as (c) an investigation of possible systematic differences between the SB detection of both methods. Generally, we set the thigh-worn inclinometer data as reference.

(a) Comparison analysis: The differences between the data from the waist- and thigh-worn monitors were compared using significance (paired *t* tests or Wilcoxon signed-rank tests) and the effect size of these differences (Cohen’s *d* or *r*). Effect sizes were considered small if *d*/*r* < 0.5, medium if 0.5 ⩽ *d*/*r* < 0.8, and large with *d*/*r* ⩾ 0.8.^28^
(b) Association analysis: The relation between SB data provided by the waist-worn and thigh-worn monitors was calculated by means of Spearman’s rank correlation coefficient rho. Reliability was measured by means of the intraclass correlation coefficient (ICC). An ICC value between 0.00 and 0.40 was considered poor, 0.40 and 0.59 was fair, 0.60 and 0.74 was good, and 0.75 and 1.00 was excellent.^29^ The data obtained by waist-worn accelerometers and thigh-worn inclinometers were visualized in Bland–Altman plots.
(c) The differences between SB parameters (total time sedentary, time in sedentary bouts), as assessed with the reference method (inclinometry at the thigh) and the comparative method (accelerometry at the waist), were calculated and used in one-tailed partial correlation analyses (corrected for the individual number of valid epochs) with participant sex, age, BMI, and individual PA level quantified by the number of steps taken within the valid epochs (mean of accelerometer and inclinometer data).

The significance level was set at *p* < 0.05. All analyses were conducted in IBM SPSS Statistics v27.0 (SPSS Inc, Chicago, IL, USA).

## Results

A total of 53 volunteers participated in this study. Participant characteristics are summarized in Table 1.

**Table 1.** Study participant characteristics.

Characteristics	Participants (*N* = 53)
Age (years)	42.0 (IQR: 25.5–56.0); range: 20.0–85.0
18–39	49.1%
40–59	35.8%
⩾60	15.1%
Sex	19 males, 34 females
Height (cm)	170.0 (IQR: 165.0–178.0); range: 158.0–189.0
Weight (kg)	75.91 ± 13.90; range: 53.0–112.0
BMI^a^	24.44 ± 3.90; range: 19.0–34.5

BMI, body mass index; IQR, interquartile ranges. Depending on the data distribution, participant characteristics are given either as means with standard deviations or as medians with IQRs.

a Body mass divided by the square of the body height (kg / m^2^).

### Validated wear time

According to all three monitors, the number of valid epochs showed a nonparametric distribution (*p* ⩽ 0.008). The waist-worn inclinometer recorded 15,544.0 (Mdn; IQR: 12,276.0–17,883.0) valid epochs. Measurements of the waist- and thigh-worn accelerometers yielded 17,490.0 (Mdn; IQR_waist_: 13,554.0–19,902.0; IQR_thigh_: 13,548.0–19,914.0) valid epochs, which equates to a difference of approximately 13%. There was no significant difference in the number of epochs acquired by the waist- and thigh-worn accelerometers (*p* = 0.517), but there was a difference between each individual accelerometer and the waist-worn inclinometer (*p* < 0.001). The wear-time data obtained with the three methods were highly correlated (rho ⩾ 0.925, *p* < 0.001). The participants wore the monitors for 4 days (Mdn; IQR: 3–4). During this time, 43.18 valid hours (Mdn; IQR: 33.76–49.76) could be recorded. The average number of valid hours per day was calculated as 11.29 ± 2.65 h.

### Comparison of SB obtained by accelerometry and inclinometry on the waist and thigh

SB was quantified by determining the times in sitting and lying postures as well as the number of corresponding 10-min bouts. Except for the time in sedentary bouts acquired by waist-worn accelerometers (*p* = 0.033), all data collected at both monitors were normally distributed (*p* ⩾ 0.065).

As part of the outlier correction, three data sets were removed from the total 212 data sets (four data sets each from 53 participants). Data from the synchronized inclinometer and accelerometer measurements are summarized in Table 2.

**Table 2.** Comparison of total sedentary time and time in sedentary bouts.

	Total sedentary time (epochs) M [95% CI]			Time in sedentary bouts (epochs) Mdn [IQR]| M [95% CI]		
Monitor position	Accelerometer	Inclinometer	*t* test; effect size	Accelerometer	Inclinometer	Wilcoxon test or *t* test; effect size
Waist	7586.73 [6900.78–8272.67]	8185.42 [7520.55–8850.29]	*t*(51) = 3.498, *p* = 0.001*; *d* = −0.485	2760.00 [1620.00–4560.00]	3941.18 [3446.30–4436.05]	*Z* = −4.918, *p* < 0.001*; *r* = −0.682
Thigh	8013.90 [7286.98–8740.83]	9092.85 [8237.19–9948.51]	*t*(52) = 3.807, *p* < 0.001*; *d* = −0.523	3753.46 [3214.13–4292.80]	5951.54 [5236.99–6666.08]	*t*(51) = −13.001, *p* < 0.001*; *r* = −1.790
*t* test; effect size	*t*(51) = −3.089, *p* = 0.003*; *d* = −0.428	*t*(52) = −4.047, *p* < 0.001*; *d* = −0.556	*t*(51) = −7.765, *p* < 0.001*; *d* = −0.731	*Z* = −5.548, *p* < 0.001*; *r* = −0.769	*t*(51) = −9.517, *p* < 0.001*; *d* = −1.219	*Z* = −6.335, *p* < 0.001*; *r* = −0.870

CI, confidence interval; IQR, interquartile ranges. Comparison of total sedentary times and times in sedentary bouts acquired by synchronous inclinometry and accelerometry at the waist and thigh. Significant differences were flagged (*), and corresponding effect size measures were calculated. The statistical comparison between the accelerometer data collected at the waist and the inclinometer data collected at the thigh is highlighted (gray background).

Regarding the overall population, total sedentary time measurements were significantly lower in accelerometers than in inclinometers (*p* < 0.001). The same was true for the assessment of sedentary bouts (*p* < 0.001). Generally, the accelerometers and inclinometers recorded more sedentary time and time in sedentary bouts when attached to the thigh (*p* < 0.001).

### Association analysis of SB obtained by accelerometry and inclinometry on the waist and thigh

Association analyses and reliability tests of the data were performed by calculating Spearman rank correlations and ICCs (Table 3).

**Table 3.** Association analyses of total sedentary time and time in sedentary bouts.

	Total sedentary time (% thigh-worn inclinometer epochs)			Time in sedentary bouts (% thigh-worn inclinometer epochs)		
Monitor position	Accelerometer	Inclinometer	SR correlation	Accelerometer	Inclinometer	SR correlation
Waist	83.44%	90.02%	rho = 0.878, *p* < 0.001*	46.37%	66.22%	rho = 0.785, *p* < 0.001*
Thigh	88.13%	100.00%	rho = 0.861, *p* < 0.001*	63.06%	100.00%	rho = 0.893, *p* < 0.001*
SR correlation	rho = 0.884, *p* < 0.001*	rho = 0.853, *p* < 0.001*	rho = 0.888, *p* < 0.001*	rho = 0.925, *p* < 0.001*	rho = 0.815, *p* < 0.001*	rho = 0.818, *p* < 0.001*
ICC (95% CI)	ICC = 0.937 (0.889–0.964), *p* < 0.001*			ICC = 0.848 (0.531–0.935), *p* < 0.001*		

CI, confidence interval; ICC, intraclass coefficient; SR, Spearman rank. Percentage deviations of the total sedentary time and time in sedentary bouts assessed by inclinometry and accelerometry at the waist and thigh were calculated and referenced to the thigh-worn inclinometer (100%). Significant correlations (SR and ICC) were flagged (*). The correlation between the accelerometer data collected at the waist and inclinometer data collected at the thigh is highlighted (gray background).

**Figure 1.**
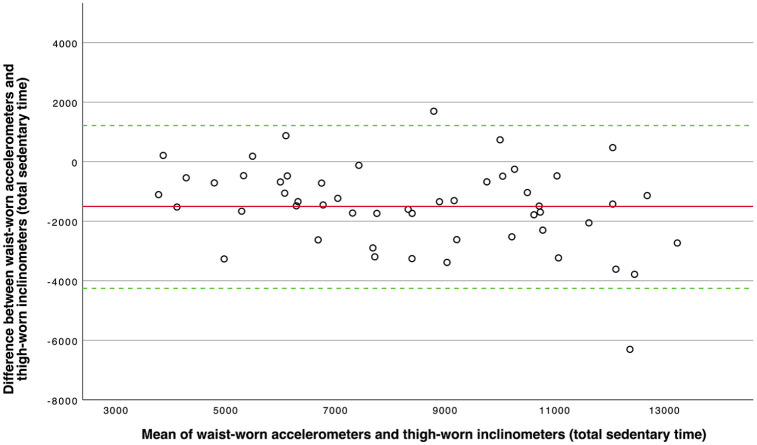
Bland–Altman plot of the total sedentary time (in epochs) assessed by waist-worn accelerometers and thigh- worn inclinometers as reference. The solid red line represents the mean difference between the two measures (−1501.63 epochs), and the dashed green lines mark the limits of agreement (low: −4234.73 epochs; high: 1231.46 epochs, i.e. ± 1.96 SD).

We also conducted a graphical analysis of waist-worn accelerometer data and thigh-worn inclinometer data for the total sedentary time (Figure 1). This analysis could not be performed for the time in sedentary bouts because the differences between both methods did not meet the criteria for normal distribution (*p* = 0.010).

### Investigation of differences in the detection of SB between accelerometry and inclinometry

**Figure 2.**
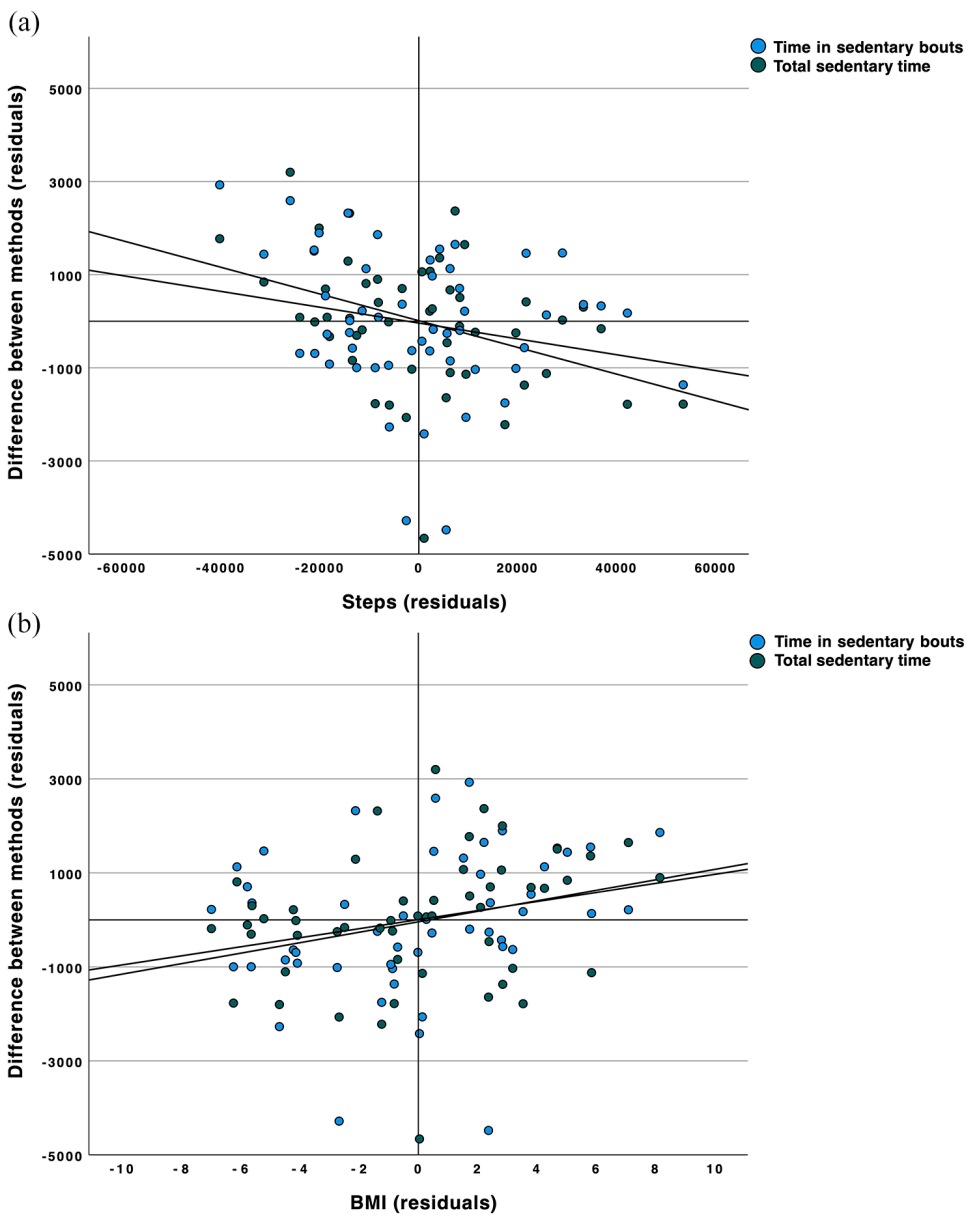
Scatter plots of the residuals (corrected for the individual number of valid epochs) of the parameters time in sedentary bouts (blue) and total sedentary time (green) with the number of steps (a) and the BMI (b) of all participants. Linear trend lines are based on data from both SB parameters.

The differences between the reference method (thigh-worn inclinometry) and the comparative method (waist-worn accelerometry) were calculated for total sedentary time [−1437.40 [−2466.95 to −574.63)] and time in sedentary bouts [−3150.00 (−3585.00 to −1515.00)] and investigated by means of partial correlation analyses (corrected for the individual number of valid epochs) with participant age, BMI, and number of steps. We found significant correlations for both parameters with BMI (*r* > 0.262, *p* < 0.032) and with number of steps (*r* > −0.240, *p* < 0.043). On average, the fewer steps taken (Figure 2(a); 10,000 steps corresponding approximately to a difference of 250 epochs) and the higher the BMI (Figure 2(b); one BMI unit corresponding approximately to a difference of 110 epochs), the smaller were the differences between the two methods investigated.

## Discussion

The core aspect of our work was to compare the waist-worn accelerometer, used in most PA studies,^30^ to the thigh-worn inclinometer as a reference. Although ActiGraph GT3X activity monitors are among the most frequently used monitors in PA and SB studies, comparative investigations of their inclinometer and accelerometer functions are still sparse, especially with the latter optimized for SB detection. Previous research often has ignored GT3X features such as measuring acceleration in all three planes (VM) and setting filters for the detection of slow movements (the LFE option), which could improve the distinction between low-intensity PA and sedentary behavior.^13^ The current study adds to the literature by comparing the performance of waist- and thigh-worn wGT3X-BT accelerometers and inclinometers with application of these features for the detection of SB in free-living adults.

Edwardson and coworkers demonstrated the detection of almost 100% of all tested sedentary activities by the inclinometer algorithm of thigh-worn ActiGraph monitors.^19^ Their findings are in line with another observational study by Steeves *et al.*^18^ showing almost perfect accuracy of the thigh algorithm for detecting sedentary activities. Skotte *et al.*^17^ also found a good performance of the thigh-worn ActiGraph monitor under free-living conditions when compared with a pressure logger to detect sitting posture.

Although thigh-worn inclinometers have obvious advantages in detecting SB, waist-worn accelerometers are still the gold standard in clinical studies for various reasons. One is the large number of existing age- and population-specific cut-point models for the evaluation of PA developed on data obtained from (and recommended for) waist-worn accelerometers.^31^ Another is that participants find it easier to wear a monitor attached to the waist compared with the thigh.^19^ To prevent slipping down the thigh during everyday activities, the elastic strap must be firmly attached or additional fixations (plasters or tape) must be used, reducing comfort even further. Most commercial monitors must be removed for any water-based activities and for sleeping, so the correct application and fixation after these activities also must be ensured. These practical disadvantages of a thigh-worn ActiGraph monitor, which can possibly result in adherence issues, have been described in detail previously.^19^

With use of the LFE on waist-worn ActiGraph accelerometers, SB detection rates of 90% and above have been achieved in direct observational studies, which is significantly higher than the detection rate with waist-worn inclinometers.^13^ The poor performance of the proprietary ActiGraph inclinometer waist algorithm has been repeatedly reported in the literature. Edwardson and coworkers investigated the recognition of several seated postures by the waist inclinometer algorithm and reported only between 46% and 70% correct detection.^19^ Our results are in contrast to these earlier findings, as we found a significantly higher detection rate (90%) of total sedentary time by the waist-worn inclinometers as opposed to accelerometers (83%). Nevertheless, the accelerometers also had an acceptable detection rate when both VM and LFE were used in free-living adults.

A fundamental problem with accelerometer-based investigation of SB is the distinction of light-intensity physical activity (LPA). SB is defined as activities that do not increase energy expenditure substantially above resting level (1.0–1.5 metabolic equivalents of task, or METs) and includes activities such as sleeping (0.95 METs), lying down (1.0–1.3 METs), watching television (1.3 METs), and doing light office work (1.5 METs).^32^ LPAs have been described as activities that result in energy expenditure at the level of 1.6–2.9 METs, such as slow walking (2.0 METs), washing dishes (1.8 METs), and cooking food (2.0 METs).^32^ By using the LFE and the VM settings, we aimed to reduce the likelihood of LPA being incorrectly interpreted as SB. With waist-worn monitors, such a misjudgment might occur during activities in which the body’s center of gravity remains at rest (e.g. any standing activity, cycling at a constant speed). In addition to good energy expenditure outcomes,^33^ thigh placement of the monitor allows for detection of such PAs with excellent accuracy.^34^ Especially in older populations for whom cycling can be a part of everyday activities (such as for commuting or shopping), this distinction is important.^35^ The same is true for orthopedic patients who use cycling to maintain joint functionality and reduce pain.^36^ For these reasons, we used the thigh-worn monitor data as a reference in our study.

Using the LFE and VM in waist-worn accelerometry achieved an acceptable approximation of the reference data from the thigh-worn inclinometers for total sedentary time but not for time in sedentary bouts (46% detection rate). In the latter case, triaxial assessment of acceleration in combination with the LFE seems to have led to significantly more aborts of sedentary bouts compared with inclinometry at the waist (20% less aborts) or accelerometry at the thigh (17% less aborts). These findings should be considered if the 10-min bout structure of SB is to be studied in addition to total sedentary times in future investigations.

In addition to comparing the detection rates of waist-worn accelerometers and thigh-worn inclinometers, we looked for factors that might influence the absolute difference in SB detection between these two methods. Remarkably, both SB parameters were significantly correlated with BMI and activity level, as determined by the number of steps taken after correcting for the individual valid epochs (as a precise equivalent to the wear time). We found that less SB (total time and time in bouts) was correctly detected in participants with lower BMI more often than in participants with higher BMI. This finding is largely in line with studies showing that individuals with obesity show more postural sway when standing still than do individuals with normal BMI.^37^ Consequently, standing posture was misidentified as sitting less often in these individuals. Wu and Mandigan proposed that an impaired plantar sensitivity with obesity might be associated with increased postural sway.^38^ However, most of our study participants did not have obesity (18.5 < BMI < 24.9), and the described correlations were weak. Regarding the step counts examined, we found that more inactive participants (those taking fewer steps) had less misidentification as sedentary. This pattern could be explained by the assumption that these participants in general perform more sedentary activities than those who are physically active and stand less frequently. Further studies in people with different PA levels could provide deeper insights.

A strength of this study is that it was conducted under real-life conditions and included a wide age range to cover a broad set of everyday behaviors (school, education, work, chores, leisure, sports). By using specific settings for the ActiGraph monitors, we applied accelerometry in a way that should improve differentiation of low PA and SB.^25^ The study also has some limitations. Most important, we relied on previous studies and used thigh-based inclinometry as the reference method. Although we demonstrated an age independence of the discrepancy between the investigated methods, the age distribution in our study population was not even. More younger than older people participated in our study, and further investigations should be carried out in older populations. Furthermore, conclusions should not be drawn about typical everyday activities (i.e. habitual PA and SB) during the periods of pandemic-related lockdowns. Even young people showed a high level of physical inactivity during these days.^39^ We have therefore largely refrained from providing average daily values or converting epochs into real-time values. Finally, we did not further investigate whether the abort of sedentary bouts in the compared methods occurred because of misidentified non-wear or misidentified standing posture. This aspect should be investigated further because time spent standing could be relevant, especially in general monitoring and in the rehabilitation process for patients with musculoskeletal diseases.

## Conclusion

SB is an important risk factor for several chronic diseases. In sedentary populations such as patients with musculoskeletal disorders, treatment-related improvements resulting in an increased ability to move around may be seen first as changes in SB rather than PA. Nevertheless, both ends of the PA spectrum should be examined together. Here, we have shown that waist-worn ActiGraph wGT3X-BT accelerometers, as used in many PA studies, also can be used to study total sedentary times if certain settings are used. Although the detection of sedentary time with waist-worn accelerometers is still significantly lower than with thigh-worn inclinometers, the results appear to be methodologically acceptable. This method cannot be used to study typical 10-min sedentary bouts, however, because it detected less than half of these correctly. Both BMI and the PA level of the participants seemed to weakly influence the agreement between waist-worn accelerometry and thigh-worn inclinometry. Researchers interested in measuring SB alongside PA by means of waist-worn ActiGraph monitors should activate LFE and VM settings and take BMI and PA level into account when establishing participant groups.

## Supplemental Material

sj-jpg-1-tab-10.1177_1759720X221079256 – Supplemental material for Measuring sedentary behavior using waist- and thigh-worn accelerometers and inclinometers – are the results comparable? Click here for additional data file. Supplemental material, sj-jpg-1-tab-10.1177_1759720X221079256 for Measuring sedentary behavior using waist- and thigh-worn accelerometers and inclinometers – are the results comparable? by Tobias Kalisch, Christoph Theil, Georg Gosheger, Thomas Ackmann, Isabell Schoenhals and Burkhard Moellenbeck in Therapeutic Advances in Musculoskeletal Disease
