# Supplementary figures and images for: Measuring sedentary behavior using waist- and thigh-worn accelerometers and inclinometers – are the results comparable?
Source: Ther Adv Musculoskelet Dis. 2022 Mar 15;14:1759720X221079256. doi: 10.1177/1759720X221079256 (PMC8928357; doi:10.1177/1759720X221079256)

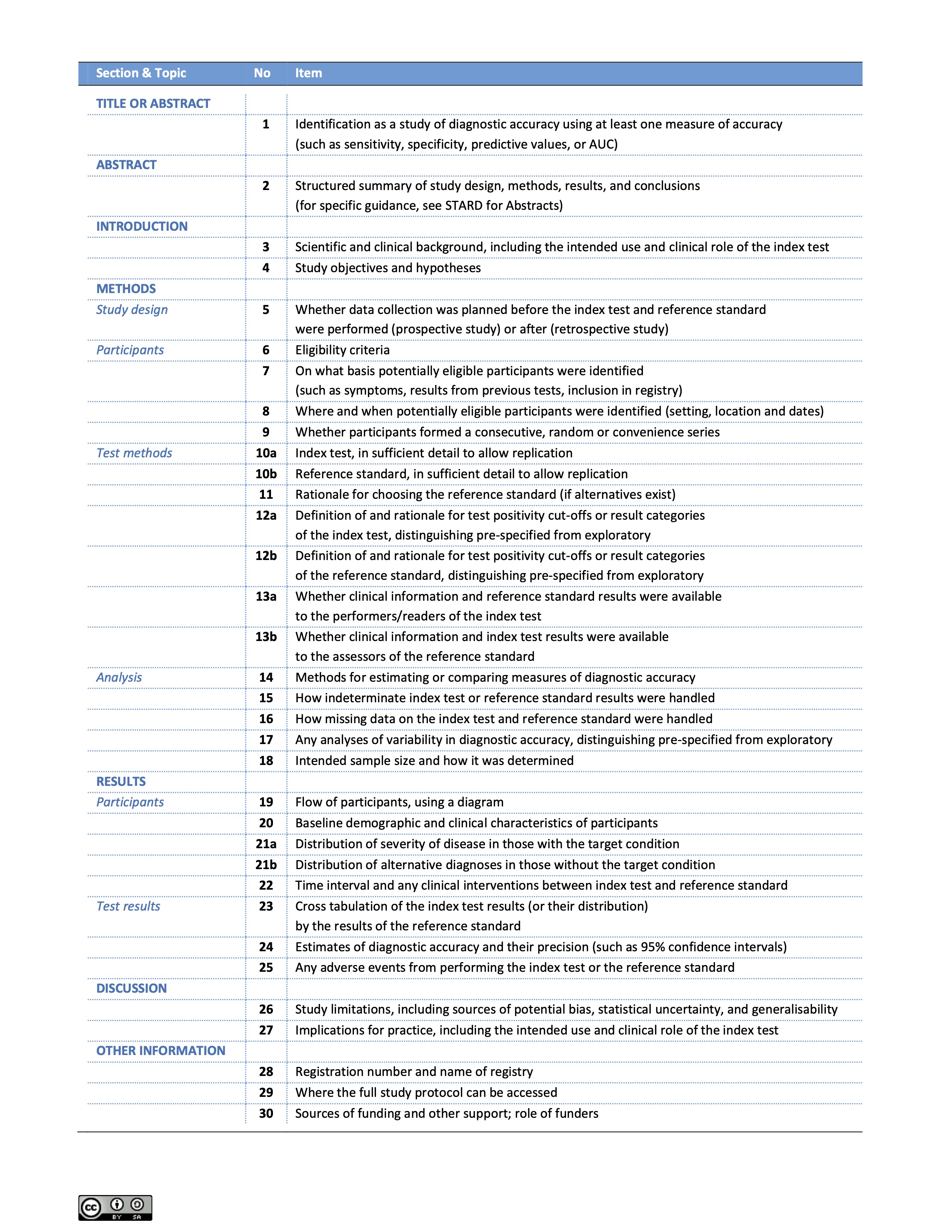

Supplement: sj-jpg-1-tab-10.1177_1759720X221079256 – Supplemental material for Measuring sedentary behavior using waist- and thigh-worn accelerometers and inclinometers – are the results comparable? [file sj-jpg-1-tab-10.1177_1759720X221079256.jpg]
